# Histopathological Growth Patterns and Survival After Resection of Colorectal Liver Metastasis: An External Validation Study

**DOI:** 10.1093/jncics/pkab026

**Published:** 2021-03-21

**Authors:** Diederik J Höppener, Boris Galjart, Pieter M H Nierop, Florian E Buisman, Eric P van der Stok, Robert R J Coebergh van den Braak, Martin J van Amerongen, Vinod P Balachandran, William R Jarnagin, T Peter Kingham, Michail Doukas, Jinru Shia, Iris D Nagtegaal, Peter B Vermeulen, Bas Groot Koerkamp, Dirk J Grünhagen, Johannes H W de Wilt, Michael I D’Angelica, Cornelis Verhoef

**Affiliations:** 1 Department of Surgical Oncology and Gastrointestinal Surgery, Erasmus MC Cancer Institute, Rotterdam, the Netherlands; 2 Department of Radiology, Radboud University Medical Center, Nijmegen, the Netherlands; 3 Department of Surgery, Memorial Sloan Kettering Cancer Center, New York, NY, USA; 4 Department of Pathology, Erasmus MC, Rotterdam, the Netherlands; 5 Department of Pathology, Memorial Sloan Kettering Cancer Center, New York, NY, USA; 6 Department of Pathology, Radboud University Medical Center, Nijmegen, the Netherlands; 7 Translational Cancer Research Unit (GZA Hospitals and University of Antwerp), Antwerp, Belgium; 8 Department of Surgery, Erasmus MC, Rotterdam, the Netherlands; 9 Department of Surgery, Radboud University Medical Center, Nijmegen, the Netherlands

## Abstract

**Background:**

After resection of colorectal cancer liver metastases (CRLM), 2 main histopathological growth patterns can be observed: a desmoplastic and a nondesmoplastic subtype. The desmoplastic subtype has been associated with superior survival. These findings require external validation.

**Methods:**

An international multicenter retrospective cohort study was conducted in patients treated surgically for CRLM at 3 tertiary hospitals in the United States and the Netherlands. Determination of histopathological growth patterns was performed on hematoxylin and eosin–stained sections of resected CRLM according to international guidelines. Patients displaying a desmoplastic histopathological phenotype (only desmoplastic growth observed) were compared with patients with a nondesmoplastic phenotype (any nondesmoplastic growth observed). Cutoff analyses on the extent of nondesmoplastic growth were performed. Overall survival (OS) and disease-free survival (DFS) were estimated using Kaplan-Meier and multivariable Cox analysis. All statistical tests were 2-sided.

**Results:**

In total 780 patients were eligible. A desmoplastic phenotype was observed in 19.1% and was associated with microsatellite instability (14.6% vs 3.6%, *P* = .01). Desmoplastic patients had superior 5-year OS (73.4%, 95% confidence interval [CI] = 64.1% to 84.0% vs 44.2%, 95% CI = 38.9% to 50.2%, *P* < .001) and DFS (32.0%, 95% CI = 22.9% to 44.7% vs 14.7%, 95% CI = 11.7% to 18.6%, *P* < .001) compared with their nondesmoplastic counterparts. A desmoplastic phenotype was associated with an adjusted hazard ratio for death of 0.36 (95% CI = 0.23 to 0.58) and 0.50 (95% CI = 0.37 to 0.66) for cancer recurrence. Prognosis was independent of *KRAS* and *BRAF* status. The cutoff analyses found no prognostic relationship between either OS or DFS and the extent of nondesmoplastic growth observed (all *P* > .1).

**Conclusions:**

This external validation study confirms the remarkably good prognosis after surgery for CRLM in patients with a desmoplastic phenotype. The extent of nondesmoplastic growth does not affect prognosis.

During the course of their disease, up to 30% of patients with colorectal cancer (CRC) present with or develop liver metastases (1). Surgical removal or ablation of CRC liver metastases (CRLM) remains the only potentially curative treatment in these patients, resulting in a 5-year overall survival (OS) of 40% to 60% (2).

At pathological examination of CRLM, 2 clinically relevant histopathological subtypes can be observed: a desmoplastic histopathological growth pattern (HGP) and a nondesmoplastic HGP. Considerable biological differences between both pathological subtypes have been demonstrated (3). The desmoplastic HGP has been associated with increased angiogenic capacity and increased infiltration of cytotoxic T cells, whereas nondesmoplastic HGP tumors mostly establish vascularization by means of cooption of preexisting hepatic sinusoidal vessels. In addition, a reduced infiltration of immune cells and increased cancer motility is observed in these tumors (4-6).

Over the years, the HGP subtypes have gained interest, and a potential impact on prognosis and the effectiveness of chemotherapy has been demonstrated (7,8). The largest patient cohort to date was published by our group, showing substantial differences in 5-year OS outcomes between patients expressing a desmoplastic HGP (78%) and patients expressing any nondesmoplastic HGP (37%) (7).

HGPs can easily be assessed on hematoxylin and eosin (H&E)–stained tissue sections, and evaluation of HGPs results in low inter- and intraobserver variability (9). Importantly, centers should be able to assess HGPs with minimal additional costs. In view of their potential clinical implications, HGPs could be an interesting biomarker to further incorporate into the clinical practice of patients with CRLM.

Before the implementation of HGPs in the clinic, external validation is required. This study therefore aims to evaluate the prognostic impact of HGPs after resection of CRLM in an international multicenter external validation cohort. Secondly, we sought to validate the optimal cutoff for HGP classification.

## Methods

### Patient Selection and Data

Patients who underwent complete surgical treatment for CRLM at either the Erasmus MC Cancer Institute (Rotterdam, the Netherlands), Memorial Sloan Kettering Cancer Center (New York, NY, USA), or Radboud University Medical Center (Nijmegen, the Netherlands) from 2000 until 2019 were potentially eligible for inclusion. Complete surgical treatment was defined as resection (with or without ablation) of all known CRLM and extrahepatic metastases if present. Patients were required to have had their primary colorectal malignancy resected as well. Patients receiving adjuvant therapies (systemic chemotherapy and/or hepatic arterial infusion pump [HAIP] chemotherapy) were excluded for 2 reasons. First, this study entails an external validation of a previously described cohort that only included patients who did not receive adjuvant therapy (7). In this external validation study, a comparable but independent cohort of patients was selected. Second, a recent article suggested modification of the effect of postoperative systemic chemotherapy by HGP, resulting in a survival benefit for the adjuvantly treated nondesmoplastic patients only (8). Exclusion of these patients ensures unbiased evaluation of the prognostic effect unaltered by postoperative therapies.

Patient demographics, clinicopathological disease characteristics, and survival data were extracted from the respective center’s prospectively maintained databases. The study adheres to the REMARK guidelines for tumor marker prognostic studies (10). Institutional ethical review and approval was obtained from the medical ethics committee of the Erasmus University Medical Center Rotterdam (MEC-2018–1743), which granted a waiver for informed consent.

### Treatment Strategy and Postoperative Course

The Erasmus MC Cancer Institute, Memorial Sloan Kettering Cancer Center, and the Radboud University Medical Center are tertiary referral centers for liver surgery. All patients with suspected CRLM were discussed by a multidisciplinary team of surgical oncologists, medical oncologists, radiation oncologists, and radiologists. Presence of limited extrahepatic disease amenable to local treatment did not preclude complete surgical treatment. Noticeable practice differences between centers exist in use of perioperative chemotherapeutic therapies. HAIP chemotherapy is commonly used at the Memorial Sloan Kettering Cancer Center and is administered frequently in selected patients (11), whereas in the Netherlands, HAIP chemotherapy is administered only within the context of randomized controlled clinical trials (12,13). Moreover, perioperative systemic chemotherapy is considered standard of care throughout the United States. In the Netherlands, guidelines advocate to administer preoperative chemotherapy only to increase resectability in patients with unresectable disease or to facilitate a parenchymal-sparing approach. Postoperative systemic chemotherapy is not advocated. Practice variation regarding perioperative systemic chemotherapy does, however, exist in the Netherlands (14).

Postoperative surveillance in all 3 centers consists of outpatient visits, serial blood serum carcinoembryonic antigen (CEA) assessments, and medical imaging by computed tomography and/or magnetic resonance imaging. Postoperative surveillance is generally scheduled every 3 to 6 months for the duration of 5 years, or longer at the patients’ discretion. In the case of recurrent disease, optimal treatment strategy is again determined by each center’s multidisciplinary team.

### Pathological Assessment

Pathological assessment of HGP was performed retrospectively on H&E sections by at least 2 trained observers simultaneously and blinded for patient characteristics and outcome. Dedicated liver pathologists were consulted when necessary. All available H&E tissue sections of all resected CRLM of each patient were assessed for HGP phenotype by light microscopy or digital evaluation of digitalized sections.

**Figure 1.**
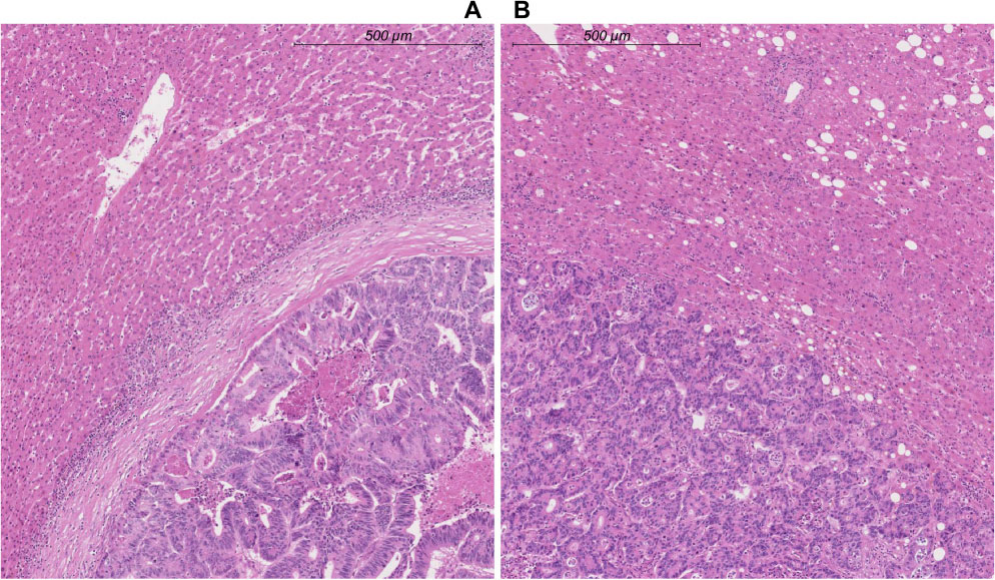
Hematoxylin and eosin–stained tissue sections of resected CRLM viewed at 5× magnification are shown with corresponding scale bars in the upper right. **A**) Hematoxylin and eosin–stained tissue section of a resected colorectal liver metastasis displaying a desmoplastic phenotype. Note the rim of desmoplastic tissue separating the tumor cells (**lower right**) from the liver parenchyma (**upper left**). **B**) Hematoxylin and eosin–stained tissue section of a resected colorectal liver metastasis displaying a nondesmoplastic phenotype. Note the absence of a desmoplastic rim and the direct contact between the tumor cells (**lower left**) and the liver parenchyma (**upper right**).

In accordance with international consensus guidelines, the tumor–liver interface was evaluated for pathological phenotype. The 3 previously described HGP phenotypes are discussed in depth in these guidelines (15). In summation, the desmoplastic phenotype is characterized by separation of tumor and liver parenchyma by a band of desmoplastic stroma (Figure 1, A). This band of desmoplastic stroma separating cancer cells from the liver parenchyma is absent in the nondesmoplastic phenotypes (Figure 1, B). Because multiple phenotypes can appear in conjunction, the relative proportion of each phenotype is estimated on each H&E section and expressed as percentage. The final patient-level score is the average of each metastasis, with equal weights assigned to discrete metastases and to individual slides within metastases. There is no minimum section requirement for HGP assessment. Sections are considered unsuitable if only a small fraction of the tumor–liver interface (<20%) is assessable, if tissue preservation quality is deemed unsuitable (eg, tear of tissue at the transition zone), or when viable tumor tissue is absent (ie, complete pathological response). Patients were classified as desmoplastic if all slides of all resected CRLM uniformly displayed a desmoplastic phenotype (ie, 100% desmoplastic; Figure 1, A) and as nondesmoplastic if any nondesmoplastic phenotype was observed in any slide of any resected CRLM (ie, <100% desmoplastic; Figure 1, B) (7). For cutoff analyses, patients were classified in subgroups according to the extent of nondesmoplastic phenotypes observed: 100% desmoplastic vs 0.1% to 33%, 33.1% to 67%, and 67.1% to 100% nondesmoplastic, respectively.

### Outcomes

OS and disease-free survival (DFS) were evaluated. OS was defined as time from surgical resection to death. DFS was defined as the time from surgical resection to cancer recurrence or death, whichever came first. Patients were censored if alive with no evidence of disease. Outcomes were additionally evaluated stratified for preoperative chemotherapy status.

### Statistical Analyses

Categorical data are reported as absolute count with corresponding percentage. Nonparametric continuous data are reported as median with corresponding interquartile range. Differences in proportions were evaluated by means of the χ^2^ test. Medians were compared by the Kruskal-Wallis test. Survival curves were estimated according to Kaplan-Meier analysis and compared by means of the log-rank test. Five-year survival estimates with corresponding 95% confidence intervals (CIs) are reported. Median follow-up for survivors was determined using the reverse Kaplan-Meier method. Univariate and multivariable Cox proportional hazards regression survival analyses were performed and reported as hazard ratios (HRs) with corresponding 95% confidence intervals. All known clinicopathological risk factors were added to the regression models. With regards to missing data, full-case analyses were performed. The proportional hazards assumption was visually assessed by plotting Schoenfeld residuals and Kaplan-Meier curves. Because data on *KRAS* and *BRAF* mutational status were available for only less than one-half of the patients, separate Cox regression models were computed with additional correction for these genetic risk factors. Cox regression models with interaction terms were created to evaluate effect modification of HGP by preoperative chemotherapy (7). All log-rank tests and Cox regression analyses were performed with center as stratification factor. The statistical significance level was set at an α of .05. All statistical tests were 2-sided and were performed using the R Project for Statistical Computing version 4.0.3 (https://www.r-project.org/) with the packages ggplot2 (v3.3.2), rms (6.0–1), survival (v3.2–7), survminer (v0.4.8), and tableone (v0.12.0).

## Results

Between 2000 and 2019, a total of 2708 consecutive patients underwent resection of CRLM at the Erasmus MC Cancer Institute (n = 1044), Memorial Sloan Kettering Cancer Center (n = 1352), or Radboud University Medical Center (n = 312) and had resection specimens suitable for pathological HGP assessment. Of these, 732 patients treated at the Erasmus MC Cancer Institute are described in our previous article (7), 582 received perioperative HAIP chemotherapy, 446 were treated with postoperative systemic chemotherapy, and 168 did not undergo complete surgical treatment, resulting in a total of 780 patients included in the current external validation study. Baseline characteristics stratified by center are reported in Supplementary Table 1 (available online). A total of 213 patients were treated at the Erasmus MC Cancer Institute, 338 at the Memorial Sloan Kettering Cancer Center, and 229 at the Radboud University Medical Center. Of the 213 newly described patients treated at the Erasmus MC Cancer Institute, 163 (76.5%) underwent surgery outside the inclusion period of the previous study (ie, after March 2015), 10 (4.7%) were additionally identified through data requests at the IT department, and for the remaining 40 (18.7%) H&E resection specimens were previously missing but have since been recovered (7). Primary tumor and CRLM clinicopathological characteristics were comparable between centers, with the exception of the number of CRLM, presence of extrahepatic disease, and the disease-free interval between resection of primary tumor and detection of liver metastasis, all being more favorable in patients treated at the Radboud University Medical Center (Supplementary Table 1, available online).

A desmoplastic histopathological phenotype was observed in 149 (19.1%) patients and was equally distributed across centers (Table 1). Approximately one-half (n = 373, 47.8%; Table 1) of all patients were treated with preoperative systemic chemotherapy, although this did differ between treatment centers (Supplementary Table 1, available online). A desmoplastic phenotype was more often found in the pretreated subpopulation: 22.7% (n = 85 of 373) vs 15.7% (n = 64 of 407) (*P* = .01). Patients with a nondesmoplastic phenotype had slightly larger CRLM (median = 3.0 vs 2.2 cm, *P* < .001), a longer disease-free interval (median = 2 vs 0 months, *P* = .03), higher preoperative serum CEA levels (median = 11.2 vs 5.3 μg/L, *P* < .001), and more often had extrahepatic disease (11.9% vs 6.0%, *P* = .04) (Table 1). Data on *KRAS*, *BRAF*, and microsatellite stability status were available for 42.3%, 37.1%, and 23.1% of patients. The mutation rate of *KRAS* (50.0% vs 43.0%, *P* = .33) and *BRAF* (4.0% vs 3.3%, *P* = .82) did not differ between patients with a desmoplastic and a nondesmoplastic phenotype, respectively. Microsatellite instability (MSI) was, however, more often seen in the desmoplastic phenotype (14.6% vs 3.6%, *P* = .01).

**Table 1.** Baseline characteristics stratified by histopathological phenotype

Characteristic	Missing, No. (%)	Desmoplastic (n = 149)	Nondesmoplastic (n = 631)	*P* ^a^
Treatment center, No. (%)				
Erasmus MC	—	45 (30.2)	168 (26.6)	.66
MSKCC	—	63 (42.3)	275 (43.6)	
Radboud UMC	—	41 (27.5)	188 (29.8)	
Median age at resection CRLM (IQR), y	—	65.0 (52.0, 72.0)	65.0 (56.0, 72.0)	.31
Sex, No. (%)				
Male	—	92 (61.7)	374 (59.3)	.58
Female	—	57 (38.3)	257 (40.7)	
ASA classification, No. (%)				
ASA I-II	4 (0.5)	87 (59.2)	377 (59.9)	.87
ASA >II	—	60 (40.8)	252 (40.1)	
Primary tumor location, No. (%)				
Left-sided	24 (3.1)	49 (34.8)	254 (41.3)	.35
Right-sided	—	41 (29.1)	166 (27.0)	
Rectal	—	51 (36.2)	195 (31.7)	
T stage, No. (%)				
pT 0–2	56 (7.2)	21 (15.7)	76 (12.9)	.39
pT 3–4	—	113 (84.3)	514 (87.1)	
N stage, No. (%)				
N0	10 (1.3)	64 (43.5)	220 (35.3)	.06
N+	—	83 (56.5)	403 (64.7)	
Median No. of CRLM (IQR)	2 (0.3)	2.0 (1.0, 3.0)	2.0 (1.0, 3.0)	.12
Median diameter of largest CRLM (IQR), cm	3 (0.4)	2.2 (1.3, 3.3)	3.0 (2.0, 4.6)	<.001
Median disease-free interval^b^ (IQR), months	11 (1.4)	0.0 (0.0, 11.8)	2.0 (0.0, 16.0)	.03
Median preoperative CEA (IQR), µg/L	65 (8.3)	5.3 (2.7, 16.4)	11.2 (4.2, 32.5)	<.001
Preoperative systemic chemotherapy, No. (%)				
No	—	64 (43.0)	343 (54.4)	.01
Yes	—	85 (57.0)	288 (45.6)	
Resection margin involved, No. (%)				
No	1 (0.1)	136 (91.3)	541 (85.9)	.08
Yes	—	13 (8.7)	89 (14.1)	
Extrahepatic disease, No. (%)				
No	—	140 (94.0)	556 (88.1)	.04
Yes	—	9 (6.0)	75 (11.9)	
*KRAS* mutational status, No. (%)				
Wild type	450 (57.7)	29 (50.0)	155 (57.0)	.33
Mutant	—	29 (50.0)	117 (43.0)	
*BRAF* mutational status, No. (%)				
Wild type	491 (62.9)	48 (96.0)	231 (96.7)	.82
Mutant	—	2 (4.0)	8 (3.3)	
Microsatellite stability status, No. (%)				
MSS	600 (76.9)	35 (85.4)	134 (96.4)	.01
MSI	—	6 (14.6)	5 (3.6)	

a Categorical variables were compared using the χ^2^ and numerical variables using the Kruskal-Wallis test (2-sided). ASA = American Society of Anesthesiologists; CEA = carcinoembryonic antigen; CRLM = colorectal liver metastasis; Erasmus MC = Erasmus MC Cancer Institute; IQR = interquartile range; MSI = microsatellite instable; MSKCC = Memorial Sloan Kettering Cancer Center; MSS = microsatellite stable; Radboud UMC = Radboud University Medical Center.

b Between resection of primary tumor and detection of CRLM.

### OS and DFS

**Figure 2.**
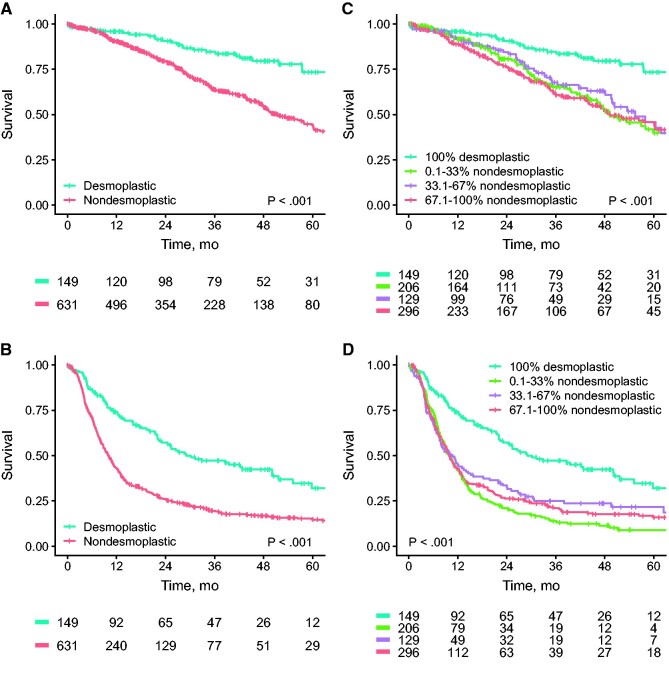
Kaplan-Meier overall survival (OS) and disease-free survival (DFS) estimates are shown. Shown are OS (**A**) and DFS (**B**) estimates of patients with a desmoplastic vs a nondesmoplastic phenotype. Shown are OS (**C**) and DFS (**D**) estimates according to the extent of nondesmoplastic growth observed. The *P* values represent the results from the 2-sided log-rank tests used to compare the survival estimates.

The median follow-up for survivors was 42 months (interquartile range = 21-66 months). During follow-up, 501 (64.2%) patients experienced recurrence and 294 (37.7%) died. Patients with a desmoplastic phenotype had statistically significantly longer OS compared with their nondesmoplastic counterparts, with 5-year OS estimates of 73.4% (95% CI = 64.1% to 84.0%) for desmoplastic vs 44.2% (95% CI = 38.9% to 50.2%) for nondesmoplastic (Figure 2, A; *P* < .001). Similar differences were observed for DFS, with 5-year estimates of 32.0% (95% CI = 22.9% to 44.7%) for desmoplastic vs 14.7% (95% CI = 11.7% to 18.6%) for nondesmoplastic (Figure 2, B; *P* < .001). The overall recurrence rate was statistically significantly lower for the patients with a desmoplastic HGP (45.6% vs 68.6%, *P* < .001). In the full-case multivariable analysis of 625 (80.1%) patients, a desmoplastic phenotype resulted in an adjusted hazard ratio of 0.36 (95% CI = 0.23 to 0.58) for OS and 0.50 (95% CI = 0.37 to 0.66) for DFS (Table 2). Considering *KRAS* and *BRAF* mutation status, 227 (29.1%) full cases were available for multivariable analysis and a desmoplastic phenotype remained independently associated with both OS (adjusted HR = 0.43, 95% CI = 0.20 to 0.92) and DFS (adjusted HR = 0.42, 95% CI = 0.25 to 0.70) (Table 3).

**Table 2.** Univariate and multivariable Cox regression analyses for overall and disease-free survival

Characteristic	Overall survival				Disease-free survival			
	Univariate		Multivariable (n = 625)		Univariate		Multivariable (n = 625)	
	HR (95% CI)	*P*	HR (95% CI)	*P*	HR (95% CI)	*P*	HR (95% CI)	*P*
Age at resection CRLM, y	1.01 (1.00 to 1.02)	.01	1.01 (1.00 to 1.02)	.13	1.00 (0.99 to 1.00)	.34	1.00 (0.99 to 1.01)	.95
ASA classification, >II vs I-II	1.26 (0.94 to 1.71)	.13	1.29 (0.90 to 1.87)	.17	1.14 (0.91 to 1.41)	.25	1.22 (0.95 to 1.57)	.12
Right-sided primary, yes vs no	1.46 (1.13 to 1.88)	.004	1.36 (1.00 to 1.86)	.05	1.05 (0.86 to 1.27)	.65	1.03 (0.82 to 1.29)	.81
T-stage, pT3-4 vs pT0-2	1.36 (0.92 to 2.00)	.12	1.28 (0.82 to 2.01)	.28	1.24 (0.95 to 1.61)	.11	1.09 (0.81 to 1.46)	.57
N-stage, N+ vs N0	1.18 (0.93 to 1.51)	.18	1.23 (0.91 to 1.66)	.18	1.29 (1.08 to 1.55)	.005	1.24 (1.01 to 1.53)	.04
Disease-free interval^a^ (cont.), mo	1.00 (0.99 to 1.01)	.65	1.00 (0.99 to 1.01)	.67	0.99 (0.99 to 1.00)	.01	0.99 (0.98 to 1.00)	.01
Number of CRLM (cont.)	1.10 (1.06 to 1.15)	< .001	1.09 (1.04 to 1.14)	<.001	1.11 (1.08 to 1.15)	<.001	1.08 (1.04 to 1.12)	<.001
Diameter of largest CRLM (cont.), cm	1.06 (1.03 to 1.10)	<.001	1.06 (1.02 to 1.11)	.006	1.06 (1.03 to 1.09)	<.001	1.05 (1.01 to 1.09)	.009
Preoperative CEA (cont.), 100 µg/L	1.01 (1.00 to 1.02)	.006	1.01 (1.00 to 1.02)	.03	1.01 (1.00 to 1.02)	.09	1.01 (1.00 to 1.02)	.24
Resection margin involved, yes vs no	1.83 (1.36 to 2.47)	<.001	1.22 (0.84 to 1.76)	.30	1.84 (1.47 to 2.31)	<.001	1.46 (1.11 to 1.92)	.007
Extrahepatic disease, yes vs no	1.63 (1.15 to 2.29)	.005	1.59 (1.05 to 2.41)	.03	1.85 (1.44 to 2.38)	<.001	2.21 (1.64 to 2.98)	<.001
Preoperative chemotherapy, yes vs no	1.25 (0.96 to 1.62)	.10	1.26 (0.93 to 1.71)	.13	1.45 (1.20 to 1.74)	<.001	1.26 (1.01 to 1.56)	.04
Desmoplastic phenotype, yes vs no	0.39 (0.27 to 0.56)	<.001	0.36 (0.23 to 0.58)	<.001	.44 (0.35 to 0.56)	<.001	0.50 (0.37 to 0.66)	<.001

a Between resection of primary tumor and detection of CRLM. ASA = American Society of Anesthesiologists; CEA = carcinoembryonic antigen; CI = confidence interval; cont. = entered as continuous variable; CRLM = colorectal liver metastasis; HR = hazard ratio.

**Table 3.** Univariate and multivariable Cox regression analyses for overall and disease-free survival including *KRAS* and *BRAF* status

Characteristic	Overall survival				Disease-free survival			
	Univariate		Multivariable (n = 227)		Univariate		Multivariable (n = 227)	
	HR (95% CI)	*P*	HR (95% CI)	*P*	HR (95% CI)	*P*	HR (95% CI)	*P*
Age at resection CRLM, y	1.01 (1.00 to 1.02)	.01	1.02 (1.00 to 1.04)	.05	1.00 (0.99 to 1.00)	.34	1.00 (0.99 to 1.01)	.99
ASA classification, >II vs I-II	1.26 (0.94 to 1.71)	.13	0.91 (0.52 to 1.61)	.75	1.14 (0.91 to 1.41)	.25	1.02 (0.71 to 1.48)	.91
Right-sided primary, yes vs no	1.46 (1.13 to 1.88)	.004	1.01 (0.59 to 1.71)	.98	1.05 (0.86 to 1.27)	.65	0.83 (0.58 to 1.19)	.32
T-stage, pT3-4 vs pT0-2	1.36 (0.92 to 2.00)	.12	1.74 (0.73 to 4.11)	.21	1.24 (0.95 to 1.61)	.11	1.48 (0.86 to 2.56)	.16
N-stage, N+ vs N0	1.18 (0.93 to 1.51)	.18	0.98 (0.58 to 1.66)	.95	1.29 (1.08 to 1.55)	.005	1.15 (0.80 to 1.67)	.45
Disease-free interval^a^ (cont.), mo	1.00 (0.99 to 1.01)	.65	0.97 (0.95 to 0.99)	.003	0.99 (0.99 to 1.00)	.01	0.99 (0.97 to 1.00)	.01
Number of CRLM (cont.)	1.10 (1.06 to 1.15)	<.001	1.03 (0.95 to 1.11)	.46	1.11 (1.08 to 1.15)	<.001	1.06 (1.00 to 1.12)	.04
Diameter of largest CRLM (cont.), cm	1.06 (1.03 to 1.10)	<.001	1.02 (0.94 to 1.11)	.56	1.06 (1.03 to 1.09)	<.001	0.99 (0.93 to 1.06)	.81
Preoperative CEA (cont.), 100 µg/L	1.01 (1.00 to 1.02)	.006	0.95 (0.83 to 1.10)	.53	1.01 (1.00 to 1.02)	.09	1.02 (0.91 to 1.15)	.71
Resection margin involve, yes vs no	1.83 (1.36 to 2.47)	<.001	1.87 (1.01 to 3.47)	.05	1.84 (1.47 to 2.31)	<.001	1.63 (1.07 to 2.46)	.02
Extrahepatic disease, yes vs no	1.63 (1.15 to 2.29)	.005	1.49 (0.81 to 2.76)	.20	1.85 (1.44 to 2.38)	<.001	2.16 (1.41 to 3.29)	<.001
Preoperative chemotherapy, yes vs no	1.25 (0.96 to 1.62)	.10	1.44 (0.82 to 2.51)	.20	1.45 (1.20 to 1.74)	<.001	0.98 (0.68 to 1.41)	.91
*KRAS* status, mutant vs wild type	1.55 (1.11 to 2.18)	.01	2.21 (1.33 to 3.65)	.002	1.33 (1.04 to 1.70)	.03	1.43 (1.03 to 1.98)	.03
*BRAF* status, mutant vs wild type	1.59 (0.58 to 4.37)	.37	3.42 (1.00 to 11.71)	.05	1.08 (0.53 to 2.23)	.83	1.03 (0.39 to 2.72)	.95
Desmoplastic phenotype, yes vs no	0.39 (0.27 to 0.56)	<.001	0.43 (0.20 to 0.92)	.03	0.44 (0.35 to 0.56)	<.001	0.42 (0.25 to 0.70)	<.001

a Between resection of primary tumor and detection of CRLM. ASA = American Society of Anesthesiologists; CEA = carcinoembryonic antigen; CI = confidence interval; cont. = entered as continuous variable; CRLM = colorectal liver metastasis; HR = hazard ratio.

When evaluating the optimal cutoff for HGP determination, no statistically significant differences in either OS or DFS were observed between patients with a 0.1% to 33%, 33.1% to 67%, and 67.1% to 100% relative presence of nondesmoplastic HGP (all *P* > .1). Patients with a desmoplastic phenotype displayed superior survival compared with all other subgroups (all *P* < .001; Figure 2, C and D). For both OS and DFS, similar results were obtained in multivariable analysis (n = 625 full cases, all *P* < .01; Supplementary Table 2, available online).

### Effect of Preoperative Chemotherapy

No statistically significant interaction between preoperative chemotherapy and HGP was observed (OS *P* = .61, DFS *P* = .64). OS and DFS differed statistically significantly between desmoplastic and nondesmoplastic HGP patients in both the chemo-naive and pretreated subpopulations.

**Figure 3.**
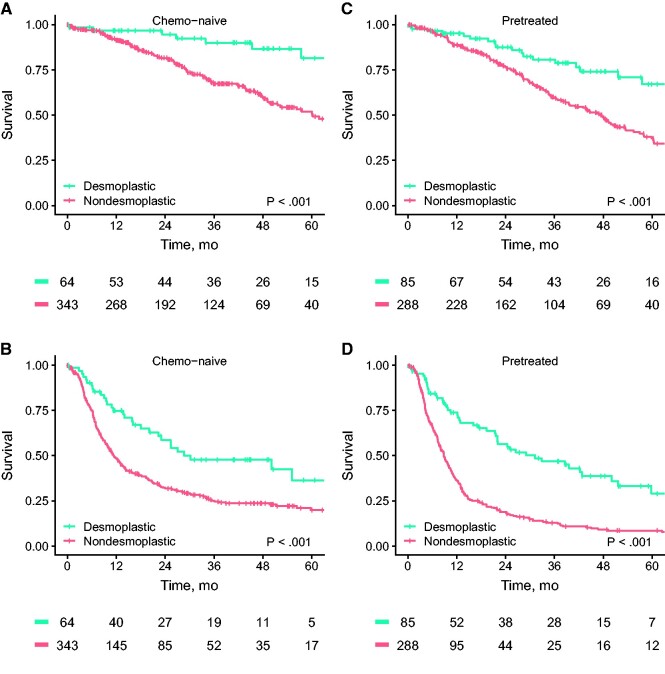
Kaplan-Meier overall survival (OS) and disease-free survival (DFS) estimates stratified by preoperative chemotherapy are shown. Shown are OS (**A**) and DFS (**B**) estimates for chemo-naive patients with a desmoplastic vs a nondesmoplastic phenotype. Shown are OS (**C**) and DFS (**D**) estimates for pretreated patients with a desmoplastic vs a nondesmoplastic phenotype. The *P* values represent the results from the 2-sided log-rank tests used to compare the survival estimates.

In chemo-naive patients, the 5-year OS estimate for a desmoplastic phenotype was 81.5% (95% CI = 68.9% to 96.5%) compared with 51.8% (95% CI = 44.4% to 60.5%) for a nondesmoplastic phenotype (Figure 3, A; *P* < .001). Again, similar differences were observed for DFS, with 5-year DFS estimates of 36.4% (95% CI = 22.6% to 58.6%) for desmoplastic vs 19.9% (95% CI = 15.0% to 26.2%) for nondesmoplastic (Figure 3, B; *P* < .001).

For pretreated patients, the 5-year OS for a desmoplastic phenotype was 67.1% (95% CI = 54.6% to 82.5%) compared with 37.1% (95% CI = 30.2% to 45.6%) for a nondesmoplastic phenotype (Figure 3, C; *P* < .001). Subsequently, the 5-year DFS was 29.0% (95% CI = 18.3% to 46.0%) for pretreated desmoplastic vs 8.6% (95% CI = 5.5% to 13.3%) for pretreated nondesmoplastic (Figure 3, D; *P* < .001).

After correction for potential confounding, a desmoplastic phenotype was associated with superior survival outcomes in both the chemo-naive (n = 352 full cases, OS: adjusted HR = 0.29, 95% CI = 0.13 to 0.65; DFS: adjusted HR = 0.53, 95% CI = 0.34 to 0.82; Supplementary Table 3, available online) and pretreated subpopulations (n = 273 full cases, OS: adjusted HR = 0.43, 95% CI = 0.23 to 0.79; DFS: adjusted HR = 0.43, 95% CI = 0.29 to 0.64; Supplementary Table 4, available online).

## Discussion

In this study, we present the results of an international multicenter external validation study on the prognostic value of HGPs after complete surgical treatment of CRLM. A desmoplastic phenotype was independently associated with superior OS and DFS outcomes in both chemo-naive and pretreated patients. Because the extent of HGP phenotypes observed can vary both within the same tumor as well as across multiple tumors in the same patient, external validation of the optimal cutoff for classification was also performed. In line with previous reports, this external validation study confirms that it is the presence of any nondesmoplastic phenotype, rather than the relative quantity, that drives prognosis.

The first report of HGPs in CRLM was published in 1991 by Morino et al. (16), and since then several reports have followed (15,17). Due to heterogeneity in histopathological assessment, cutoffs, and terminology, formal meta-analysis of the available data is not possible, but most studies demonstrate favorable outcomes in patients with a predominant desmoplastic phenotype (17). The largest study to date was published by our group and reported a 5-year OS of 78% in chemo-naive patients with a desmoplastic HGP (7). In this study, we observed a 5-year OS of 73.4% in all patients with a desmoplastic phenotype and a comparable 5-year OS of 81.5% within the chemo-naive subpopulation. In line with these results, lower recurrence rates and superior DFS were seen in patients with a desmoplastic phenotype, reflecting the remarkably good cancer-related outcomes in these patients with metastatic CRC. In addition, our study is the first to our knowledge to investigate the prognostic impact of HGPs in light of *KRAS* and *BRAF* mutational status. Although data on these genetic risk factors were available for only approximately 40% of patients, no association between the histopathological phenotype and mutations in either of these genes was observed; after correction for these genetic risk factors, a desmoplastic phenotype was still independently associated with good OS and cancer-free survival.

To standardize assessment of HGPs, international consensus guidelines have been established (15). In these guidelines, classification of HGP is based on predominance, with an advocated cutoff value of 50%. Both our previous article and the current external validation study—which represent the 2 largest studies to date—demonstrate that predominance of a distinct HGP is irrelevant. Superior survival outcomes were observed only in patients with a uniform desmoplastic phenotype. In the patients with any observed nondesmoplastic growth, the extent of this observation does not seem to bear any prognostic consequences. We therefore deem reappraisal of the current guidelines for HGP assessment necessary; classification of HGPs in CRLM should be based on the presence or absence of nondesmoplastic growth.

Besides implications for HGP assessment and postoperative prognosis, this observation is also interesting from a cancer biology perspective because it suggests that HGPs can be regarded as a binary biological switch. Although this article does not provide a clear indication for the actual underlying process, in the 23% of patients with available data, we did observe a statistically significant association between MSI and a desmoplastic phenotype. Because of their genetic hypermutability, MSI tumors express more mutational neoantigens, which can become targets for T cells (18,19). The more potential immune targets are present, the more likely an effective antitumor response can be elicited (19). This is why MSI tumors are thought to form metastases less often and why MSI represents the only indication for systemic immunotherapy in metastatic CRC so far (20,21). Because MSI tumors accounted for only 15% of patients with a desmoplastic phenotype in our study, a desmoplastic HGP could reflect more a state of (hepatic) anticancer immunity. This is supported by several other studies that demonstrated that a desmoplastic phenotype was associated with an enrichment of immune cells in the tumor microenvironment, specifically CD8+ T cells (5, 6). One could therefore hypothesize that a nondesmoplastic histopathological phenotype, observed in however small a quantity, may be a reflection of the tumor’s intrinsic or obtained ability to evade the anticancer immune response. Our study is, however, at serious risk of selection bias regarding availability of MSI status, and validation should therefore be pursued as well as research into the other biological and immunological aspects of these histopathological phenotypes.

Preoperative chemotherapy was administered in approximately one-half of the patients in this validation cohort. It has been suggested that response to chemotherapy might induce misclassification of HGP type, which could limit the applicability of HGPs in patients receiving preoperative chemotherapy (7). In our previous study, no statistically significant impact of HGPs in pretreated patients was found in multivariable OS analysis. Although this study also found a diminished adjusted hazard ratio for OS in pretreated patients, a desmoplastic phenotype remained associated with superior survival after correction for confounders. The results of this external validation study are promising to increase the applicability of this biomarker, because administration of preoperative chemotherapy is standard of care in many countries.

Many reports evaluating HGPs are now available, most of which demonstrate relevant prognostic and clinical implications (6,7,9,15,17,22-30). In addition, the effect of HGPs on survival (adjusted HR = 0.36) is considerable, underlining its importance. We therefore feel that application in clinical practice should be pursued. An important step would be incorporation of the desmoplastic and nondesmoplastic phenotypes in the standard pathological report after resection of CRLM. This can be done on standard H&E slides with excellent intraobserver agreement (9), limited resources, and minimal additional time or medical costs required. If included in the standard pathological assessment, this prognostic information becomes readily available for clinicians and could be incorporated in individual counseling of patients. Herein, a desmoplastic phenotype could be considered a marker for good prospects regarding survivorship. In addition, efforts should be made to determine whether the effectiveness of postoperative chemotherapy can be predicted by the HGP phenotype. Buisman et al. (8) showed no benefit of postoperative chemotherapy in patients with a desmoplastic HGP, but validation of these results is needed. Being a postoperative pathology-based biomarker, the impact on preoperative decision making is absent for now. Cheng et al. (31) showed that preoperative assessment of HGPs can, however, be done on imaging with an area under curve of over 0.9. When validated and optimized for use in clinical practice, HGPs could also be assessed and used in preoperative medical decision making.

This study presents the largest cohort investigating the prognostic impact of HGPs after resection of CRLM currently available and validates findings from previous studies. Nevertheless, the study has its limitations, which are mostly related to its retrospective nature. An important limitation also remains the limited data on established genetic risk factors, because *KRAS* and *BRAF* mutation status was available for only less than one-half of patients (32). Many of the patients in this study were treated before the introduction of standard molecular testing, and in earlier years mutation status was determined only in patients with disease recurrence for choice of palliative systemic chemotherapy regimens, underscoring the risk of selection bias. Nevertheless, in those patients with data on *KRAS* and *BRAF* no association or impact on prognosis was seen. In addition, correction for sidedness of the primary tumor, which can be considered a weak proxy for mutational status (33-37), also did not diminish the prognostic value of a desmoplastic phenotype. Similar risk for selection bias exists regarding MSI status, which we found to be associated with a desmoplastic phenotype. Although our study therefore does assess HGPs in light of *KRAS*, *BRAF*, and MSI status, in-depth genetic association studies on these histopathological phenotypes are needed to limit potential bias, confirm our findings, and investigate other CRC driver genes.

In conclusion, this study validates the prognostic impact of a desmoplastic phenotype in a large international multicenter cohort of surgically treated CRLM patients. We were able to confirm that patients with a desmoplastic phenotype have superior survival outcomes compared with patients with any observed nondesmoplastic phenotype. The extent of nondesmoplastic growth does not affect prognosis. These data show that HGP harbor important prognostic value, warranting implementation in clinical practice.

## Funding

None.

## Notes

**Role of the funder:** Not applicable.

**Disclosures:** The authors have nothing to disclose.

**Author contributions:** DJH and BG had full access to all data and take full responsibility for all analyses presented. The study was conceived by DJH, BG, PMHN, FEB, DJG, MID, and CV. Histopathological examination and clinical data collection was performed by DJH, BG, PMHN, FEB, EPvdS, RRJCvdB, MJvA, MD, JS, IDN, and PBV. All authors facilitated in the data collection and data analysis, and critically appraised and revised the manuscript.

## Data Availability

The data underlying this article cannot be shared publicly due to US and Dutch laws governing patient privacy and personal data. The authors nevertheless encourage further research and collaborations into the histopathological growth patterns. As such the data will be shared on reasonable request to all senior authors of the 3 participating centers and under the provisions of data transfer agreements.

## Supplementary Material

pkab026_Supplementary_Data
