# Supplementary material for: Histopathological Growth Patterns and Survival After Resection of Colorectal Liver Metastasis: An External Validation Study
Source: JNCI Cancer Spectr. 2021 Mar 21;5(3):pkab026. doi: 10.1093/jncics/pkab026 (PMC8152695; doi:10.1093/jncics/pkab026)
Supplement: pkab026_Supplementary_Data [file pkab026_supplementary_data.pdf]

Supplementary Table 1. Baseline characteristics stratified by treatment center

		missing (%)	Erasmus MC n = 213 (%)	MSKCC n = 338 (%)	Radboud UMC n = 229 (%)	p-value
Age at resection CRLM - (median [IQR])			65.0 [58.0, 71.0]	61.0 [52.0, 72.0]	67.0 [60.0, 73.0]	<0.001
Gender	Male		146 (69)	175 (52)	145 (63)	<0.001
	Female		67 (31)	163 (48)	84 (37)	
ASA classification	ASA I-II	4 (1)	187 (88)	93 (28)	184 (81)	<0.001
	ASA >II		25 (12)	244 (72)	43 (19)	
Primary tumor location	Left-sided	24 (3)	87 (42)	132 (41)	84 (38)	0.10
	Right-sided		48 (23)	101 (31)	58 (26)	
	Rectal		74 (35)	90 (28)	82 (37)	
T-stage	pT 0-2	56 (7)	28 (13)	31 (11)	38 (17)	0.15
	pT 3-4		182 (87)	256 (89)	189 (83)	
N-stage	N0	10 (1)	86 (41)	118 (35)	80 (35)	0.36
	N+		124 (59)	215 (65)	147 (65)	
Number of CRLM - (median [IQR])		2 (0)	2.0 [1.0, 4.0]	2.0 [1.0, 3.0]	1.0 [1.0, 3.0]	<0.001
Diameter of largest CRLM in cm - (median [IQR])		3 (0)	2.8 [1.9, 4.5]	2.8 [2.0, 4.5]	2.8 [1.9, 4.3]	0.67
Disease-free interval in months* - (median [IQR])		11 (1)	0.0 [0.0, 11.0]	0.0 [0.0, 19.0]	6.0 [0.0, 18.0]	0.004
Preoperative CEA in µg/L - (median [IQR])		65 (8)	11.3 [4.5, 33.5]	8.6 [3.4, 25.9]	10.0 [3.8, 30.0]	0.28
Preoperative systemic chemotherapy	No		135 (63)	103 (30)	169 (74)	<0.001
	Yes		78 (37)	235 (70)	60 (26)	
Resection margin involved	No	1 (0)	179 (84)	294 (87)	204 (89)	0.35
	Yes		33 (16)	44 (13)	25 (11)	
Extrahepatic disease	No		190 (89)	283 (84)	223 (97)	<0.001
	Yes		23 (11)	55 (16)	6 (3)	
KRAS mutational status	Wildtype	450 (58)	24 (50)	131 (56)	29 (60)	0.59
	Mutant		24 (50)	103 (44)	19 (40)	
BRAF mutational status	Wildtype	491 (63)	43 (96)	198 (97)	38 (95)	0.75
	Mutant		2 (4)	6 (3)	2 (5)	
Microsatellite stability status	MSS	600 (77)	54 (96)	60 (91)	55 (95)	0.42
	MSI		2 (4)	6 (9)	3 (5)	
Histopathological phenotype	Desmoplastic		45 (21)	63 (19)	41 (18)	0.66
	Non-desmoplastic		168 (79)	275 (81)	188 (82)	

* Between resection of primary tumor and detection of CRLM
Abbreviations in alphabetical order: ASA: American Society of Anesthesiologists; CEA: carcinoembryonic antigen; CRLM: colorectal liver metastasis; Erasmus MC: Erasmus MC Cancer Institute; IQR: interquartile range; MSI: microsatellite instable; MSKCC: Memorial Sloan Kettering Cancer Center; MSS: microsatellite stable; Radboud UMC: Radboud University Medical Center.

Supplementary Table 2. Uni- and multivariable Cox regression models of cut-off analyses for overall and disease-free survival

	Overall survival				Disease-free survival			
	Univariable		Multivariable (n=625)		Univariable		Multivariable (n=625)	
	HR [95%CI]	p-value	HR [95%CI]	p-value	HR [95%CI]	p-value	HR [95%CI]	p-value
Age at resection CRLM - years	1.01 [1.00-1.02]	0.01	1.01 [1.00-1.02]	0.11	1.00 [0.99-1.00]	0.34	1.00 [0.99-1.01]	0.94
ASA classification - >II vs I-II	1.26 [0.94-1.71]	0.13	1.27 [0.88-1.84]	0.20	1.14 [0.91-1.41]	0.25	1.21 [0.94-1.56]	0.13
Right-sided primary - yes vs no	1.46 [1.13-1.88]	0.004	1.37 [1.00-1.88]	0.05	1.05 [0.86-1.27]	0.65	1.04 [0.82-1.31]	0.75
T-stage - pT3-4 vs pT0-2	1.36 [0.92-2.00]	0.12	1.28 [0.82-2.00]	0.28	1.24 [0.95-1.61]	0.11	1.09 [0.81-1.46]	0.57
N-stage - N+ vs N0	1.18 [0.93-1.51]	0.18	1.23 [0.91-1.66]	0.19	1.29 [1.08-1.55]	0.005	1.25 [1.01-1.54]	0.04
Disease-free interval* (cont.) - months	1.00 [0.99-1.01]	0.65	1.00 [0.99-1.01]	0.71	0.99 [0.99-1.00]	0.01	0.99 [0.98-1.00]	0.01
Number of CRLM (cont.)	1.10 [1.06-1.15]	<0.001	1.09 [1.04-1.14]	<0.001	1.11 [1.08-1.15]	<0.001	1.08 [1.04-1.12]	<0.001
Diameter of largest CRLM (cont.) - cm	1.06 [1.03-1.10]	<0.001	1.07 [1.02-1.11]	0.005	1.06 [1.03-1.09]	<0.001	1.05 [1.01-1.09]	0.008
Preoperative CEA (cont.) - 100 µg/L	1.01 [1.00-1.02]	0.006	1.01 [1.00-1.02]	0.03	1.01 [1.00-1.02]	0.09	1.01 [1.00-1.02]	0.24
Resection margin involved - yes vs no	1.83 [1.36-2.47]	<0.001	1.23 [0.85-1.78]	0.27	1.84 [1.47-2.31]	<0.001	1.45 [1.10-1.91]	0.008
Extrahepatic disease - yes vs no	1.63 [1.15-2.29]	0.005	1.62 [1.07-2.45]	0.02	1.85 [1.44-2.38]	<0.001	2.19 [1.62-2.95]	<0.001
Preoperative chemotherapy - yes vs no	1.25 [0.96-1.62]	0.10	1.27 [0.93-1.73]	0.13	1.45 [1.20-1.74]	<0.001	1.25 [1.00-1.55]	0.05
Desmoplastic phenotype	Reference		Reference		Reference		Reference	
0.1-33% non-desmoplastic	2.53 [1.68-3.82]	<0.001	2.90 [1.75-4.82]	<0.001	2.49 [1.89-3.27]	<0.001	2.07 [1.51-2.85]	<0.001
33.1-67% non-desmoplastic	2.15 [1.37-3.36]	<0.001	2.30 [1.33-3.97]	0.003	2.02 [1.48-2.74]	<0.001	1.82 [1.27-2.60]	0.001
67.1-100% non-desmoplastic	2.80 [1.91-4.11]	<0.001	2.89 [1.77-4.73]	<0.001	2.24 [1.72-2.91]	<0.001	2.07 [1.51-2.82]	<0.001

* Between resection of primary tumor and detection of CRLM

Abbreviations in alphabetical order: ASA: American Society of Anesthesiologists; Cont.: entered as continuous variable; CEA: carcinoembryonic antigen; CRLM: colorectal liver metastasis.

Supplementary Table 3. Uni- and multivariable Cox regression analyses for overall and disease-free survival within the chemo-naive subpopulations

	Overall survival				Disease-free survival			
	Univariable		Multivariable (n=352)		Univariable		Multivariable (n=352)	
	HR [95%CI]	p-value	HR [95%CI]	p-value	HR [95%CI]	p-value	HR [95%CI]	p-value
Age at resection CRLM - years	1.02 [1.00-1.04]	0.02	1.02 [1.00-1.05]	0.05	1.00 [0.99-1.01]	0.78	1.00 [0.99-1.02]	0.47
ASA classification - >II vs I-II	1.57 [0.97-2.55]	0.07	1.47 [0.87-2.48]	0.15	1.27 [0.92-1.76]	0.14	1.24 [0.88-1.75]	0.22
Right-sided primary - yes vs no	1.65 [1.12-2.44]	0.01	1.36 [0.86-2.15]	0.19	1.22 [0.93-1.59]	0.16	1.12 [0.82-1.53]	0.47
T-stage - pT3-4 vs pT0-2	2.07 [1.11-3.86]	0.02	1.75 [0.89-3.43]	0.11	1.58 [1.07-2.33]	0.02	1.41 [0.93-2.13]	0.10
N-stage - N+ vs N0	1.32 [0.92-1.89]	0.14	1.41 [0.92-2.16]	0.11	1.27 [0.99-1.63]	0.06	1.32 [1.00-1.76]	0.05
Disease-free interval* (cont.) - months	1.00 [0.99-1.01]	0.43	1.00 [0.99-1.02]	0.51	0.99 [0.99-1.00]	0.15	0.99 [0.98-1.00]	0.10
Number of CRLM (cont.)	1.08 [1.00-1.17]	0.06	1.08 [0.99-1.17]	0.09	1.16 [1.09-1.22]	<0.001	1.14 [1.07-1.22]	<0.001
Diameter of largest CRLM (cont.) - cm	1.07 [1.01-1.13]	0.02	1.08 [1.01-1.16]	0.03	1.04 [0.99-1.09]	0.11	1.06 [1.00-1.13]	0.05
Preoperative CEA (cont.) - 100 µg/L	1.05 [1.01-1.11]	0.03	1.04 [0.98-1.10]	0.24	1.02 [0.97-1.07]	0.43	0.99 [0.93-1.06]	0.88
Resection margin involved - yes vs no	1.32 [0.76-2.27]	0.32	1.25 [0.67-2.34]	0.49	1.63 [1.12-2.38]	0.01	1.63 [1.07-2.48]	0.02
Extrahepatic disease - yes vs no	1.61 [0.85-3.04]	0.15	1.63 [0.76-3.49]	0.21	2.01 [1.34-3.03]	<0.001	1.69 [1.07-2.69]	0.03
Desmoplastic phenotype - yes vs no	0.34 [0.18-0.64]	<0.001	0.29 [0.13-0.65]	0.003	0.49 [0.33-0.72]	<0.001	0.53 [0.34-0.82]	0.005

Supplementary Table 4. Uni- and multivariable Cox regression analyses for overall and disease-free survival within the pre-treated subpopulations

	Overall survival				Disease-free survival			
	Univariable		Multivariable (n=273)		Univariable		Multivariable (n=273)	
	HR [95%CI]	p-value	HR [95%CI]	p-value	HR [95%CI]	p-value	HR [95%CI]	p-value
Age at resection CRLM - years	1.01 [1.00-1.02]	0.06	1.01 [0.99-1.03]	0.22	1.00 [0.99-1.01]	0.49	1.00 [0.98-1.01]	0.70
ASA classification - >II vs I-II	1.17 [0.80-1.72]	0.42	1.01 [0.60-1.71]	0.97	1.05 [0.79-1.41]	0.73	1.16 [0.79-1.70]	0.45
Right-sided primary - yes vs no	1.54 [1.09-2.18]	0.01	1.46 [0.93-2.30]	0.10	0.95 [0.72-1.25]	0.69	0.92 [0.65-1.31]	0.65
T-stage - pT3-4 vs pT0-2	1.01 [0.60-1.71]	0.96	1.01 [0.52-1.95]	0.98	0.88 [0.61-1.27]	0.50	0.76 [0.48-1.19]	0.23
N-stage - N+ vs N0	1.06 [0.76-1.49]	0.73	1.14 [0.72-1.80]	0.59	1.19 [0.92-1.55]	0.18	1.18 [0.84-1.65]	0.34
Disease-free interval* (cont.) - months	1.00 [0.98-1.01]	0.38	0.98 [0.97-1.00]	0.07	1.00 [0.99-1.00]	0.23	0.99 [0.98-1.00]	0.09
Number of CRLM (cont.)	1.11 [1.06-1.16]	<0.001	1.09 [1.03-1.16]	0.004	1.09 [1.05-1.13]	<0.001	1.05 [1.00-1.10]	0.05
Diameter of largest CRLM (cont.) - cm	1.07 [1.02-1.12]	0.006	1.06 [0.99-1.13]	0.10	1.06 [1.03-1.10]	<0.001	1.05 [1.00-1.11]	0.05
Preoperative CEA (cont.) - 100 µg/L	1.01 [1.00-1.02]	0.03	1.01 [1.00-1.02]	0.05	1.01 [1.00-1.01]	0.18	1.00 [0.99-1.01]	0.53
Resection margin involved - yes vs no	2.11 [1.45-3.09]	<0.001	1.31 [0.79-2.18]	0.29	1.89 [1.41-2.53]	<0.001	1.73 [1.17-2.55]	0.006
Extrahepatic disease - yes vs no	1.67 [1.11-2.52]	0.01	2.23 [1.31-3.81]	0.003	1.60 [1.16-2.20]	0.004	2.62 [1.73-3.98]	<0.001
Desmoplastic phenotype - yes vs no	0.41 [0.26-0.65]	<0.001	0.43 [0.23-0.79]	0.007	0.37 [0.27-0.52]	<0.001	0.43 [0.29-0.64]	<0.001

* Between resection of primary tumor and detection of CRLM
Abbreviations in alphabetical order: ASA: American Society of Anesthesiologists; Cont.: entered as continuous variable; CEA: carcinoembryonic antigen; CRLM: colorectal liver metastasis.
